# Genetics of craniosynostosis: review of the literature

**Published:** 2009

**Authors:** Alexandru Vlad Ciurea, Corneliu Toader

**Affiliations:** * First Neurosurgical Department, ‘Bagdasar-Arseni’ Clinical Emergency Hospital, Bucharest; ** Neurosurgical Department, National Institute of Neurology and Neurovascular Diseases, Bucharest; *** “Carol Davila”University of Medicine and Pharmacy Bucharest, Romania

**Keywords:** craniosynostosis, craniofacial deformities, genetic, children, Crouzon syndrome, Apert syndrome, fibroblast growth factor receptor, TWIST

## Abstract

Craniosynostosis represents a defection of the skull caused by early fusion of one or more cranial sutures. The shape alteration of the cranial vault varies, depending on the fused sutures, so that compensatory growth occurs in dimensions not restricted by sutures. Craniosynostosis can be divided into two main groups: syndromic and nonsyndromic. Nonsyndromic craniosynostosis is typically an isolated finding that is classified according to the suture(s) involved. Syndromic craniosynostosis is associated with various dysmorphisms involving the face, skeleton, nervous system and is usually accompanied by developmental delay.

In the last 15 years, research on craniosynostosis has progressed from the description of gross abnormalities to the understanding of the genetic basis of certain cranial deformities. Mutations in the genes encoding fibroblast growth factor receptors 1, 2 and 3 (FGFR-1, FGFR-2, FGFR-3), TWIST and MSX2 (muscle segment homebox 2) have been identified in certain syndromic craniosynostosis. The molecular basis of many types of syndromic craniosynostosis is known and diagnostic testing strategies will often lead to a specific diagnosis. Although the clarification of a genetic lesion does not have a direct impact on the management of the patient in many cases, there is a significant benefit in providing accurate prenatal diagnosis.

This review summarizes the available knowledge on cranisynostosis and presents a graduated strategy for the genetic diagnosis of these craniofacial defects.

## Introduction

Craniosynostosis is the premature fusion of calvarial bones leading to an abnormal head shape. Craniosynostosis is a common malformation occurring in 1 of 2000 live births. The craniosynostosis syndromes are clinically heterogeneous with overlapping features, and, sometimes, an accurate diagnosis is difficult to be made. The molecular basis of many types of syndromic craniosynostosis is known, and diagnostic testing strategies will often lead to a specific diagnosis. Although the clarification of a genetic lesion does not have a direct impact on the management of the patient in many cases, there is a significant benefit in providing accurate prenatal diagnosis [**1**].

**Fig. 1:**
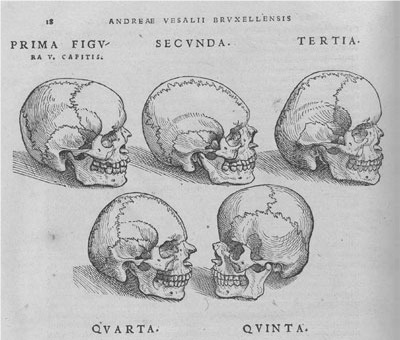
From *De Humani Corporis Fabrica*, Andreas Vesalius, 1543. Skulls depicting absence of cranial sutures and abnormal skull shapes. Reproducted with permission of [**2**]: Cunningham et al.: Syndromic craniosynostosis: from history to hydrogen bonds, *Orthod Craniofacial Res* **10**, 2007; 67–81.

Craniosynostosis was known from ancient history. The Egyptian Pharaoh Tutankhamen and his father, Amenophis the Fourth, presented a form of dolichocephaly. Although clinical descriptions of craniosynostosis date back to Hippocrates and Galen, it is generally accepted that the first historical reference to craniosynostosis was made by Mestrius Plutarchus (46–127AD) [**2**]. Known in English as Plutarch, this historian and biographer described the Athenian statesman Pericles (495 BC–429 BC) as being „squill headed” in 75 AD. Approximately 1500 years later, Andreas Vesalius characterized specific skull shapes associated with the absence of cranial sutures in *De Humani Corporis Fabrica* (**Fig. 1**) [**3**]. The recent identification of two Precolumbian skulls with sagittal synostosis (dated between 6000 and 250BC) confirm that craniosynostosis is an ancient disorder of humans [**4**].

Normal development of cranial sutures depends on the coordinated morphogenetic interactions of the osteogenic front’s component as judged by the relative spatial orientation of each front within the suture complex and regulation of the ultimate expression of the osteogenic potential.

**Suture morphogenesis**

Aberrations in either of these processes could, in theory, result in suture agenesis or synostosis. Research made in the last decades, at the cellular and molecular level, tried to highlight the process of cranial osteodifferentiation as revealed by the synthetic activities of the osteoblast and, eventually, the relative roles played by individual matrix constituents in bone formation and suture morphogenesis [**6**]. Calvarial sutures are articulations along the margins of adjacent intra-membranous bones. The function of the suture is to permit molding at the birth canal, adjustment for the expanding brain, and absorption of mechanical trauma in childhood. Fontanelles are formed at the junctional boundaries of the cranial sutures where larger areas of connective tissue occur without underlying the bone. The fusion of sutures that accompany normal development leads to closure of the posterior (formed at the junction of sagittal and lambdoid sutures) and anterior (formed at the junction of sagittal, coronal, and frontal sutures) fontanelles by 3 and 20 months, respectively. Craniosynostosis is the result of premature ossification and fusion of the skull sutures and generally results in the alteration of the shape of the cranial vault and/or premature closure of the fontanelles.

**Fig. 2:**
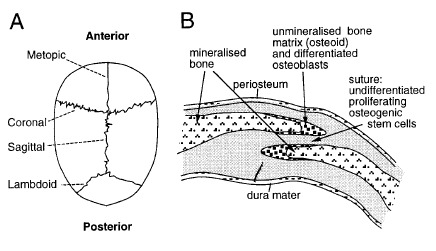
Normal cranial suture development. **(A)** View of child’s skull from above, showing position of the major sutures. Coronal craniosynostosis leads to a short, broad skull; conversely, sagittal synostosis leads to a long, narrow skull. **(B)** Diagrammatic cross section through coronal suture. The skull bones overlap slightly. In craniosynostosis, the narrow space separating the bones is obliterated. Reproducted with permission from [**4**] Wilkie AOM: Craniosynostosis: genes and mechanisms, Human Molecular Genetics, 1997, Vol. **6**, No. **10** Review.

In humans, the mineralization of the cranial vault mostly occurs directly from the derived membrane of the paraxial mesoderm, proceeding outwards from several ossification centres after approximately 13 weeks of embryonic development [**4**]. At approximately 18 weeks, these mineralizing bone fronts meet and sutures are induced along the lines of approximation. Subsequently, the skull enlarges by appositional growth at the suture with deposition of premineralised bone matrix (osteoid) along the suture margins. The major cranial sutures are shown in **Fig. 2A**. Premature fusion of one or more of these sutures (craniosynostosis) prevents further growth along the margin; excessive growth at other sutures leads to skull distortion. The suture itself is anatomically a simple structure (**Fig. 2B**), comprising the two plates of bone separated by a narrow space containing immature, rapidly dividing osteogenic stem cells, a proportion of which are recruited to differentiate from osteoblasts and make new bone. [**4**]

**Fig. 3:**
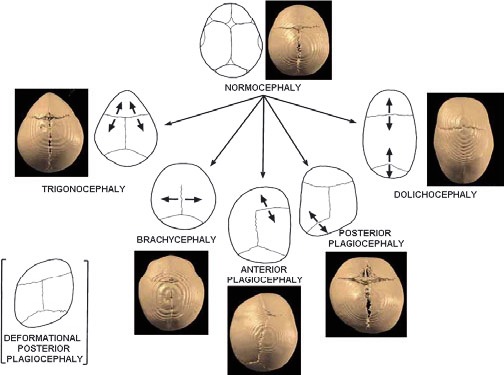
Schematic presentation of the calvarial sutures and the skull deformities resulting from the synostosis of a particular suture. The arrows indicate the direction of skull growth. 3D-CT images of patients illustrate types of craniosynotosis. Reproducted with permission from [**7**] Boyadjiev SA: Genetic analysis of non-syndromic craniosynostosis, *Orthod Craniofacial Res* **10**, 2007; 129–137).

The alteration in shape of the cranial vault varies with the fused sutures, so that compensatory growth occurs in dimensions not restricted by sutures (**Fig. 3**). Normally, the skull grows in planes perpendicular to the sutures, but premature fusion forces growth in a plane parallel to the closed suture [**5**].

**Classifications**

Craniosynostosis may be classified depending on:

- the number of the involved sutures: simple (involving 1 suture) or complex (involving two or more sutures);

- etiology: primary (caused by an intrinsic defect in the suture) or secondary (premature closure of normal sutures because of another medical condition such as deficient brain growth);

- isolated (occurring without other anomalies) or syndromic (accompanied by other dysmorphic features or developmental defects).

All subclassifications of craniosynostosis can be genetic.

The following are the frequencies of the various sutures involved: 

• sagittal: 40% to 58%, etiology unknown;

• coronal: 20% to 29%, estimated one third caused by single-gene mutations;

• metopic: 4% to 10%, etiology unknown;

• lambdoidal: 2% to 4%, etiology unknown [**5**].

## Classification Based on Suture Involvement

**Sagittal**

Synostosis of the sagittal suture is the most frequent simple craniosynostosis and shows a strong male predominance (male: female ratio of 3.5:1). It accounts for 40% to 58% of all cases of craniosynostosis and has an estimated birth prevalence of 1.9 to 2.3 per 10,000 live births. Only 2% of the cases involving sagittal synostosis are thought to be familial. The fusion of the sagittal suture results in an increase of the anterior-posterior diameter of the skull called dolichocephaly or scaphocephaly. Twinning, increased parity, maternal smoking and intrauterine head constraint have been suggested as risk factors [**5**].

**Coronal**

Unilateral (anterior plagiocephaly) or bilateral (brachycephaly) fusion of the coronal suture is the second most common form of craniosynostosis. It accounts for 20–30% of all NSC cases and has an estimated incidence of 0.8–1 in 10.000 live births with 60–75% of those affected being females. About 8–10% of coronal synostosis patients have a positive family history. Unilateral coronal craniosynostosis should be clinically differentiated from positional plagiocephaly. Progressive frontal plagiocephaly can also result from the fusion of the frontosphenoidal or frontozygomatic sutures this mandating detailed 3D-CT imaging with 1mm cuts of the basilar coronal ring sutures in plagiocephalic patients with open coronal sutures. High proportions of familial cases and the suggested increase in average paternal age may indicate a stronger genetic component to coronal than to sagittal NSC. Special clinical attention and/or targeted FGFR3 (P250R) and TWIST analyses are needed to differentiate true coronal NSC from mild cases of Muenke and Saethre–Chotzen syndromes [**7**].

**Metopic**

Metopic synostosis is associated with trigonocephaly and has an incidence of 1 per 10,000 to 15,000 births with a male preponderance. It accounts for approximately 14% of all craniosynostosis cases and has a male: female ratio of 3.3:1 [**8**]. Trigonocephaly presents itself as being an isolated anomaly present in approximately 70% of the cases and having the recurrence risk of 3.2%. Several syndromes are associated with metopic synostosis, including Baller-Gerold, Jacobsen (including 11q24.1 deletion), chromosome 9p deletion and Opitz C syndrome; therefore, genetic testing of such patients is warranted, including a karyotype analysis, possible microarray analysis for microdeletions, and/or selected mutation analysis depending on the clinical scenario. An important, perhaps life-threatening consequence of metopic craniosynostosis is the Chiari I malformation that has been found in 30% of patients with metopic ridges [**9**].

**Lambdoid**

**Fig. 4:**
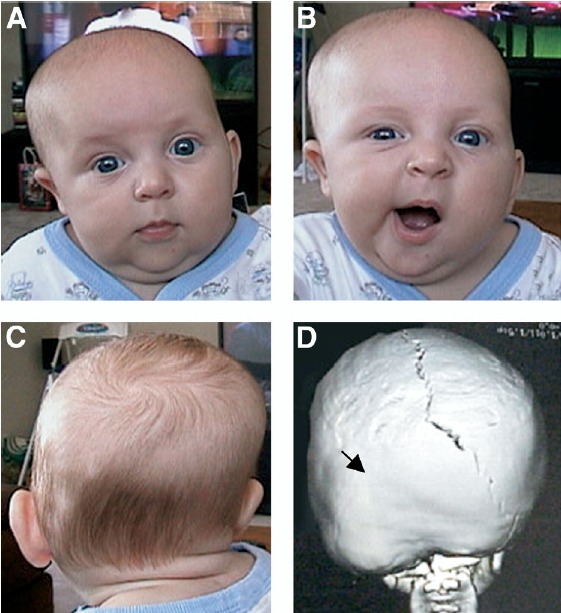
Posterior plagiocephaly caused by lambdoidal synostosis. (A and B) Frontal and (C) posterior views of an infant with left lambdoidal synostosis; the 3D-CT reconstruction (D, posterior view) shows premature fusion of the left lambdoidal suture (arrow). Note the facial asymmetry of the patient secondary to the lambdoidal syostosis (panel B) that does not occur in positional plagiocephaly. Reproducted with permission from: Kimonis Virginia, Gold JA, Hoffman T, Panchal J, Boyadjiev S: Genetics of Craniosynostosis, *Semin Pediatr Neurol* **14**:150-161, 2007. [**5**].

Lambdoid craniosynostosis is the least common type of craniosynostosis, accounting for only 2% to 4% of all NSC. In bilateral lambdoid synostosis, the entire occipital region is flattened and widened. Most cases of lambdoid craniosynostosis are unilateral and result in asymmetric posterior plagiocephaly that needs to be differentiated from positional plagiocephaly. These two conditions pose a significant diagnostic dilemma that requires careful clinical and radiologic differentiation and different therapeutic approaches (**Fig. 4**) [**5**]. 3D-CT has proved to be the most useful modality for documenting lambdoid fusion because lambdoid sutures are not readily visualized on skull radiographs and routine computed tomography study might not detect partial sutural fusion [**10**]. In cases of severe and progressive plagiocephaly with open lambdoid sutures, synostoses of the asterion region or the mendosal suture must be excluded by a detailed 3D-CT scan. Associations of lambdoid synostosis with intrauterine constraint, preterm labor and male gender have been suggested.

**Multiple**

Craniosynostosis of multiple sutures accounts for approximately 5% of craniosynostosis. It is clinically separated into two groups: 2-suture disease (including bicoronal synostosis) and complex craniosynostosis with fusion of more than two sutures. Patients with 2-suture fusion typically have a similar long-term neurodevelopmental outcome to those with single-suture involvement, except for a higher rate of reoperation [**11**]. Complex craniosynostosis frequently causes increased ICP and is associated with developmental delay and a high rate of reoperation. In patients with complex craniosynostosis, involving both coronal and sagittal sutures, increased ICP was present in two thirds, whereas three quarters had Chiari I, an anomaly on magnetic resonance imaging. Numerous patients with elevated ICP secondary to craniosynostosis had normal funduscopy, indicating the need for a computed tomography diagnostic scan when evaluating these patients.

Complex synostosis involving all sutures is known as pansynostosis, which is frequently associated with a genetic syndrome. Kleeblattschädel or cloverleaf skull deformity is a phenotypic description seen in severe pansynostosis, causing protrusion of the brain through the open anterior and parietal fontanelles. This deformity is associated with multiple syndromes, including thanatophoric dysplasia, Crouzon, Pfeiffer, and Carpenter syndromes [**5**].

## Syndromic Craniosynostosis

More than 180 different syndromes involve craniosynostosis [**5**]. The following clinical descriptions are intended to cover the more common and well-characterized forms of craniosynostosis.

**Apert syndrome**

**Fig. 5:**
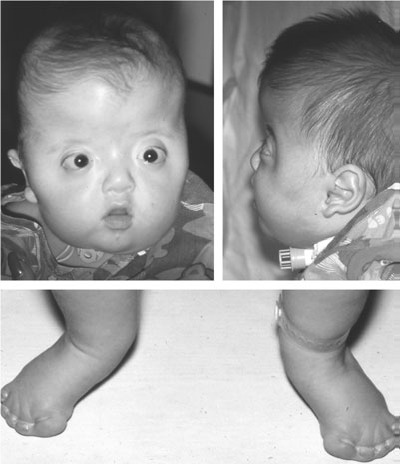
Classic features of Apert syndrome including turribrachycephaly, proptosis, midface hypoplasia and syndactyly. Airway compromise, because of midface hypoplasia, necessitated tracheostomy. Reproducted with permission from Cunningham et al. [**2**]

The French pediatrician Dr. Eugène Charles Apert (1868–1940), described in 1906 nine cases of syndactyly associated with acrocephaly. Now known as Apert syndrome, this condition is characterized by coronal craniosynostosis, syndactyly, symphalangism (fusion between the phalanges of the digits), radiohumeral fusion and variable mental retardation (**Fig. 5**). Although it is usually considered to have a very consistent phenotype, large case series have demonstrated wide variation in the craniofacial phenotype and neurocognitive outcome [**12**]. Apert syndrome demonstrates an autosomal dominant mode of inheritance and is associated with advanced paternal age. Management of children with Apert syndrome includes surgical correction of the craniosynostosis, midfacial hypoplasia and syndactyly. In 1995, Wilkie et al. [**13**] identified FGFR2 (S252W) and FGFR2 (P253R) mutations in each of the 40 unrelated cases. In addition, four distinct mutations in FGFR2 (S252W, P253R, and two Alu insertions) have been identified as causative in this debilitating condition [**2**].

**Crouzon syndrome**

**Fig. 6:**
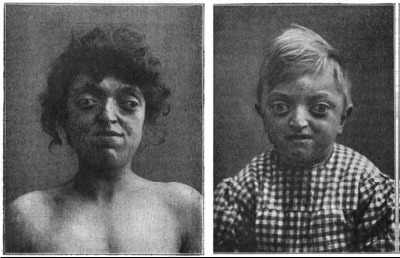
Original cases described by Crouzon demonstrating prognathism, maxillary hypoplasia, exophthalmos, papilledema, and divergent strabismus. Reproducted with permission from Cunningham et al. [**2**]

In 1912, the neurologist Louis Edouard Octave Crouzon (1854–1918) described a 29-year-old woman with prognathism, maxillary hypoplasia, exophthalmos, papilledema, hypermetropia, occipital headaches and divergent strabismus (**Fig. 6**) [**14**]. In addition, he described her 3-year-old son who had a similar facial appearance with frontal bossing, bilateral exophthalmia, strabismus and papillary oedema and recognized the hereditary nature of this disorder. Now known as Crouzon syndrome, this form of hereditary craniosynostosis also demonstrates wide phenotypic variability.

The most frequent manifestations of Crouzon syndrome include coronal craniosynostosis with variable involvement of other calvarial sutures, brachycephaly, frontal bossing, proptosis, hypertelorism, strabismus, maxillary hypoplasia, mandibular prognathism, atresia of the external auditory canals, premature calcification of stylohyoid ligament, Chiari I malformation, hydrocephalus and mental retardation. In 1994, Reardon et al. [**12**] described mutations in the third Ig domain of FGFR2 as the cause of Crouzon syndrome.

**Muenke syndrome**

**Fig. 7:**
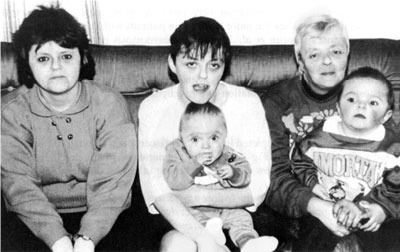
Original family with autosomal dominant coronal synostosis reported by Glass et al. Note the mild midfacial hypoplasia, hypertelorism, and downslanting palpebral fissures. This family later found to harbor the FGFR (3P250R) mutation of Muenke syndrome.Reproducted with permission from Cunningham et al. [**2**]

In 1994 Glass et al. [**16**] described a family of five affected individuals with a variable autosomal dominant phenotype including premature coronal sutural synostosis accompanied by a mild midfacial hypoplasia, hypertelorism, downslanting palpebral fissures, beaking of the nose and brachydactyly (**Fig. 7**). Often inaccurately described as a syndrome that was based on molecular rather than phenotypic findings, we now know that the family described by Glass harbored the FGFR (3P250R) mutation of what is now commonly known as Muenke syndrome [**17**]. The identification of the P250R mutation in FGFR3 occurring in 20 unrelated families served as the definition of this craniosynostosis syndrome [**18**]. Since its initial description, the phenotype of Muenke syndrome has evolved to include unilateral or bilateral coronal craniosynostosis, brachydactyly, thimble-like middle phalanges, coned epiphyses, carpal and tarsal fusions, sensorineural hearing loss, Klippel–Feil anomaly and infrequent cognitive impairment [**2**].

In 1976, Drs. Jackson, Weiss and their coauthors described a large Amish kindred in which 138 members were reported to have an unusual spectrum of craniofacial and foot anomalies [**21**]. Like Crouzon syndrome, the craniofacial features in the original kindred included hypertelorism, proptosis, midface hypoplasia, and acrocephaly. The clinical foot anomalies included a broad and medially deviated great toe, partial cutaneous syndactyly of second and third toes, broad and short metatarsals. Radiographs of the affected individuals’ feet revealed fusion of the tarsal and metatarsal bones, broad short first metatarsals, and broad proximal phalanges. As described in their manuscript ‘The only consistent manifestation of the gene observed has been some abnormality in the clinical or radiologic appearance of the feet’ [**21**]. In 1994, Jabs et al. [**19**] identified an FGFR2 (A344G) mutation in affected individuals of the original Amish kindred. A wide variability in expression of the Jackson–Weiss syndrome phenotype in this kindred was subsequently described in 2001 [**22**].

**Pfeiffer syndrome**

In 1964, a German geneticist from the University of Münster, Rudolf Arthur Pfeiffer, described the Pfeiffer syndrome as ‘Dominant erbliche krocephalosyndaktylie‘ (dominant hereditary acrocephalosyndactyly) [**23**]. The main characteristics are craniosynostosis, midface deficiency, unusually broad, short, great toes, broad thumbs and variable brachydactyly [**24**].

Pfeiffer syndrome can be further delineated into three subgroups, although there is an overlap particularly between types 2 and 3. Type 1 is the most common and has a good prognosis. Impairment of intellect is unlikely without other associated malformations such as loss of hearing or hydrocephalus. Type 2 is more severe and associated with a poor prognosis. It occurs at birth or prenatally by cloverleaf skull, severe ocular proptosis, broad thumbs and great toes with medial deviation. Additional malformations may include choanal stenosis or atresia, laryngotracheal abnormalities, elbow ankylosis/synostosis, hydrocephalus, seizures, and intellectual disability. Type 3 has a similar facial appearance to type 2 but without cloverleaf skull. Intellectual disability is also common. The majority of the patients with Pfeiffer syndrome present mutations in FGFR2, although a small number have also been identified in FGFR1 (<5%). Inheritance is autosomal dominant.

**Crouzon syndrome with acanthosis nigricans (Crouzonodermoskeletal syndrome)**

Acanthosis nigricans associated with a phenotype resembling Crouzon syndrome was first described by Helene Ollendorff Curth in 1971 in an article about benign, malignant and syndromic acanthosis nigricans [**25**].

**Fig. 8:**
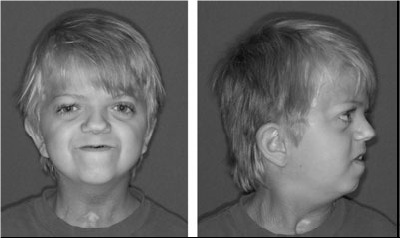
Craniofacial and skin findings in Crouzonodermoskeletal syndrome. After successful cranioplasty to treat Kleeblatschaedel skull deformity, he has residual midfacial hypoplasia. Note periorbital and perioral acanthosis nigricans. This patient was found to have the classic FGFR3 (A319E) mutation of Crouzono-dermoskeletal syndrome. Reproducted with permission from Cunningham et al. [**2**].

In 1985, Dr. Lorraine Suslak proposed that the association of the three rather rare conditions (Crouzon syndrome, acanthosis nigricans and odontogenic tumors) suggested that this was a single gene disorder but did not address whether this was a rare feature of Crouzon syndrome or a distinct entity (**Fig. 8**) [**26**].

In 1995, Meyers et al. [**27**] described a novel A391E substitution in the transmembrane domain of FGFR3 only 11 amino acids from the Gly380Arg mutation of achondroplastic dwarfism. There have been no reports of the FGFR3 (A391E) mutation in cases of Crouzon syndrome in the absence of acanthosis nigricans. Since the original description by Curth in 1971, approximately 37 additional cases have been described in literature. Although it appears to be one of the rarest forms of syndromic craniosynostosis, the phenotypic similarities with Crouzon syndrome may lead to under diagnosis.

**Saethre–Chotzen syndrome**

**Fig. 9:**
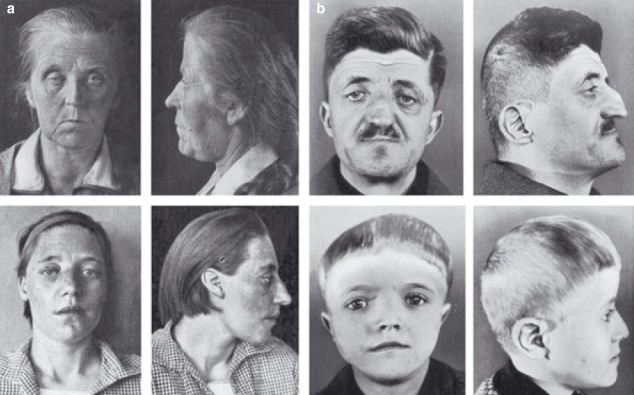
Original cases described by (a) Saethre and (b) Chotzen in 1931 and 1932, respectively. Note the low frontal hairline, ptosis, facial asymmetry, and deviated nasal septum. Reproducted with permission from Cunningham et al. [**2**]

In 1931, Haakon Saethre, a Norwegian psychiatrist, described a woman with craniosynostosis, a low frontal hairline, facial asymmetry, deviated nasal septum, defects of the vertebral column, brachydactyly, fifth finger clinodactyly and partial soft tissue syndactyly of the second to third fingers and second to fourth toes (**Fig. 9a**) [**28**]. One year later a German psychiatrist Dr F. Chotzen of Breslau, described similar craniofacial malformations in a father and two sons (**Fig. 9b**) [**29**]. The combination of these reports suggested a specific phenotype and heritability. Previously called acrocephalosyndactyly type III, classic Saethre–Chotzen syndrome (SCS) is characterized by unilateral or bilateral coronal synostosis, facial asymmetry (particularly in individuals with unicoronal synostosis), ptosis, ocular hypertelorism, a low frontal hairline, maxillary hypoplasia, a characteristic appearance of the ear (small pinna with a prominent crus) and short stature.

Syndactyly of fingers two and three of the hand and hallux valgus are variably present. Although mild-to-moderate developmental delay and mental retardation have been reported, the vast majority of individuals with point mutations are of normal intelligence. The risk for developmental delay in individuals with deletions involving the TWIST1 gene is approximately 90%, or eightfold greater than in individuals with intragenic mutations. Less common manifestations of SCS include parietal foramina, vetebral fusions, radioulnar synostosis, cleft palate, duplicated distal hallucal phalanx, and congenital heart malformations. Although phenotypic similarities between SCS and Muenke syndrome have been noted, the classic features of low hairline, ptosis, small ears, and two to three syndactyly make the SCS phenotype quite distinct.

**Craniofrontonasal Dysplasia**

This is an X-linked dominant disorder that affects females more severely [**30**]. Features include coronal synostosis with brachycephaly and features of frontonasal dysplasia including hypertelorism, anterior widow’s peak, downslanting palpebral fissures, clefting of the nasal tip, and occasionally cleft lip and palate. Other finger and joint anomalies, abnormal clavicles, and raised scapulae are associated with Sprengel deformity. Longitudinally grooved fingernails are characteristic of this disorder. Mutations in EFNB1 located at Xq12 are causative.

## Molecular genetics and developmental pathogenesis of craniosynostosis

In the past decade, significant progress has been made in understanding the genetic basis of certain craniosynostosis syndromes, with mutations in the fibroblast growth factor (FGF) signaling a pathway playing a central role [**5**]. The most common and well-characterized cases of craniosynostosis have been caused by mutation in the FGFR1 (fibroblast growth factor receptor 1), FGFR2, FGFR3, TWIST and MSX2 (muscle segment homebox 2) genes [**4**].

**Mutations in the fibroblast growth factor receptor (FGFR) genes**

**Fig. 10:**
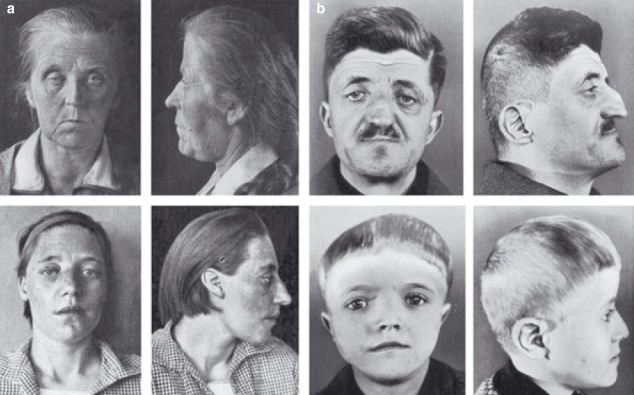
The position of craniosynostosis syndrome mutations on FGFR 1 to 3. FGFRs each contain 3 immunoglobulin-like domains (Ig I-III), a single transmembrane domain (TM), and 2 tyrosine kinase domains (TK1-2). The location of various mutations is shown. Different syndromes may result from identical mutations in *FGFR2*, whereas mutations in *FGFR1* and *FGFR2* are able to cause the same clinical phenotype. Adapted with permission from Kimonis Virginia et al. [**5**].

FGFs are a family of at least 22 known signaling molecules that function to regulate cell proliferation, differentiation and migration through a variety of complex pathways. They are important in angiogenesis, wound healing, limb development, mesoderm induction/patterning, neuronal differentiation, malignant transformation and skeletogenesis. They act through the fibroblast growth factor receptors (FGFRs), a family of 4 tyrosine kinase receptors. The FGFRs share the general structure of a split cytoplasmic tyrosine kinase domain, a transmembrane domain, and an extracellular domain that contains 3 immunoglobulin-like repeats (**Fig. 10**) [**5**].

The fibroblast growth factor receptor mutations seen in craniosynostosis syndromes can be divided into four categories: 1) immunoglobulin like domain II–III linker region mutations, 2) immunoglobulin-like domain III mutations, 3) transmembrane domain mutations, and 4) tyrosine kinase domain mutations [**2**].

Gain-of-function mutations in FGFR1 to 3 have been associated with Pfeiffer, Apert, Crouzon, Beare-Stevenson, Jackson-Weiss and Muenke syndromes. All the above syndromes are most frequently characterized by bicoronal craniosynostosis or cloverleaf skull, distinctive facial features, and variable hand and foot findings. Interestingly, identical FGFR2 mutations (e.g. C278F, G298P, and C342T) have been found in patients carrying the diagnosis of Crouzon, Pfeiffer, and Jackson-Weiss craniosynostosis syndromes, suggesting that these entities may represent a clinical spectrum with possible genetic modifiers [**31**-**33**]. It has also been shown that the same clinical phenotype can result from mutations in different genes, such as cases of Pfeiffer syndrome with mutations in FGFR1 and FGFR2 [**32**] suggesting functional redundancy among different FGFR molecules.

If one considers the craniofacial phenotype resulting from the linker region mutations of FGFR1 (P252R) (Type I Pfeiffer), FGFR2 (P253R), (S252W) (Apert), and FGFR3 (P250R) (Muenke), one can see obvious similarities. Each principally involves the coronal suture and, when bilateral, it results in a remarkably similar skull phenotype (turribrachycephaly) that is distinct from the skull phenotype of classic Crouzon syndrome [**2**].

Kinetic ligand-binding studies and X-ray crystallography of linker region mutations in FGFR1 (P252R), FGFR2 (P253R), (S252W) and FGFR3 (P250R) demonstrate that these mutations result in increased ligand affinity and altered specificity [**34**–**37**]. Interestingly, the FGFR2c linker domain mutations of Apert syndrome result in enhanced binding of FGF7 and FGF10, both mesenchymally expressed ligands of the FGFR2b isoform and important in limb development. The FGFR1c and FGFR3c mutations do not enhance binding of either FGF7 or FGF10 but rather enhance the binding affinity of FGF9, which is expressed very well in the calvaria [**38**]. Taken together these data suggest that the mutations associated with Apert syndrome affect affinity for the mesodermally expressed by FGF7 and FGF10, resulting in autocrine signaling. In contrast, the mutations of Type I Pfeiffer and Muenke syndrome enhance the affinity for FGF9 and a natural ligand for both FGFR1c and FGFR3c expressed in the epithelium. During early human limb bud development, FGFR1 and FGFR2 are expressed in both the ectoderm and mesoderm, while FGFR3 is undetectable [**2**].

Molecular modeling suggested that these mutations disrupt intramolecular disulfide bonds in the IgIII domain allowing the bonding of intermolecular disulfide and constitutive activation. Taken together these data provide strong evidence of aberrant intermolecular disulfide bonds between unpaired cysteine residues, as a molecular consequence of FGFR2 mutations resulting in Crouzon, Jackson-Weiss and Pfeiffer phenotypes regardless of the involvement of a cysteine residue. These molecular findings place these disorders in an entirely different mechanistic category from the mutations of Type I Pfeiffer, Apert and Muenke syndromes, each associated with altered ligand-binding specificity and/or affinity [**2**].

In addition to the disorders of the FGFR-related craniosynostosis spectrum, mutations in several transcription factors have also been implicated in syndromic craniosynostosis [**5**].

**Mutation in the TWIST genes**

The majority of patients with Saethre-Chotzen syndrome (SCS) present loss-of-function mutations in transcription factor TWIST1 [**39**, **40**] which is thought to negatively regulate FGFR1, 2 and 3 and the osteogenic transcription factor Runx2. In the past, over 100 distinct mutations in the TWIST1 gene have been found to cause SCS. These include nucleotide substitutions (missense and nonsense), deletions, insertions, duplications, and complex rearrangements [**41**, **42**–**44**]. Nonsense mutations that preclude translation of the DNA binding domain and the HLH domain have been identified from the 5¢ end of the coding sequence to the end of the HLH motif. Functional haplo-insufficiency, either through deletion, inhibition of dimerization, or impaired DNA binding appears to be a unifying molecular mechanism underlying the pathogenesis of SCS [**45**]. The migratory populations of cephalic neural crest cells are the origin of the membranous bones of the skull (frontal, parietal, and squamosal), their intervening sutures, overlying dermis, and underlying dura mater. Suture mesenchyme (intrasutural mesenchyme) and the osteogenic fronts demonstrate high levels of Twist1 expression [**46**]. With regard to osteoblast development, the Twist-box domain of Twist1 binds to the DNA-binding domain of Runx2, reversibly inhibiting its function [**47**]. Runx2 is a major bone regulatory transcription factor that increases the expression of osteocalcin through interaction with the vitamin D receptor [**48**]. It is presumed that the derepression of RUNX2 in the presence of TWIST1 mutations is directly related to the pathogenesis of SCS. *In vitro* culture of human osteoblasts naturally harboring TWIST1 mutations demonstrated significant reduction in proliferation, alkaline phosphotase activity, RUNX2 expression and a trend toward increased mineralization, suggesting that loss-of-function mutations of TWIST1 lead to reduced proliferation and enhanced differentiation [**49**].

**Mutations in MSX2 genes**

Boston-type craniosynostosis caused by a gain of function mutation in MSX2 (muscle segment homebox 2) has been described in a single family with variable phenotype ranging from metopic ridging to cloverleaf skull and digital abnormalities [**50**]. Animal models indicate that MSX2 is expressed in osteoblasts adjacent to the calvarial sutures and loss of function mutations in this gene leads to skull ossification defects in humans, consistent role of this gene in bone formation [**51**].

**Genetic screening of patients with craniosynostosis**

Although the clarification of a genetic lesion does not have a direct impact on the patient’s management in many cases, there is a significant benefit in providing accurate prenatal diagnosis. Genetic counselors are also able to offer better risk estimates of recurrences to non-manifesting carriers and their extended family members as well as for affected patients of reproductive age [**1**].

Diagnostic strategy for craniosynostosis testing includes a sequential analysis of recurrent mutations (**Table 1**), followed by a selective sequencing. This algorithm makes testing of cranio-synostosis disorders more efficient and cost-effective [**1**]. The genetic screening is suitable for those patients who accomplished minimal clinical diagnostic criteria for common craniosynostosis disorders [**1**] (**Table 2**).

**Genetic Counseling**

The majority of craniosynostosis are autosomal dominant. Due to variable expressivity, the identification of a mutation in an affected individual should be followed by parental testing. In more severe types of craniosynostosis, the de novo mutation rate is high. In autosomal dominant types of craniosynostosis, mutation carriers have a 50% risk of passing the affected gene to their offspring. Negative parental mutation testing still leaves a small (<1%) risk of recurrence because of potential gonadal mosaicism [**5**].

**Table 1:** **Molecular Testing of recurrent mutations** (Adapted with permission from [**5**]).

Disorder	Gene (% responsible)	Gene (% responsible) Mutations	Mutation Detection Rate
Pfeiffer	FGFR2 (>95%) FGFR1 (<5%)	Several	67%
Apert	FGFR2 (100%)	Ser252Trp, Pro253Arg	>98%
Crouzon	FGFR2 (100%)	Several	>50%
Crouzon with acanthosis	FGFR3 (100%)	Ala391Glu	100%
Muenke	FGFR3 (100%)	Pro250Arg	100%
Saerthre Chotzen	TWIST1	Several mutations & deletions	46% to 80%

**Table 2:** **Minimal Clinical Diagnostic Criteria for Common Craniosynostosis ** (Adapted with permission from [**1**]).

Condition	Minimal diagnostic criteria
Apert syndrome	Craniosynostosis, midface hypoplasia, symmetrical syndactyly of hands and feet
Crouzon syndrome	Craniosynostosis, maxillary hypoplasia, shallow orbits, ocular proptosis, normal extremities
Muenke syndrome	Unilateral or bilateral coronal synostosis, absent or minimal hand/foot anomalies
Pfeiffer syndrome	Craniosynostosis, high forehead, maxillary hypoplasia, mild syndactyly of hands and/or feet, broad thumbs and/or great toe
Saerthre-Chotzen syndrome	Brachycephaly/plagiocephaly +/- evidence for craniosynostosis, high forehead, facial asymmetry, maxillary hypoplasia, brachydactyly, partial cutaneous syndactyly in some cases, thumb/great toe anomalies

Prenatal testing strategies include chorionic villus sampling (typically at 10-14 weeks gestation) or amniocentesis (typically at 16-18 weeks gestation). Preimplantation genetic diagnosis is a valuable option available for those who have been identified as carriers of the mutation but are interested in ensuring that their children are unaffected, without making an abortion in the event of a positive prenatal diagnosis. Molecular testing enables the selection of genetically normal embryos to use in vitro fertilization [**5**].

## Conclusions

In the last century, we have advanced from the first clinical description of Apert syndrome to the description of the FGFR, TWIST and MSX2 mutations. The basic science that has driven the molecular discoveries of the past 15 years will continue to advance our basic understanding of the molecular causes of these conditions. In the future, it becomes increasingly important to focus on researches that will have an impact on the treatment of these disorders.
